# Editorial of the Special Issue Titled “Drug Candidates for the Prevention and Treatment of Cardiovascular Diseases”

**DOI:** 10.3390/ph17040417

**Published:** 2024-03-26

**Authors:** Nikolaos P. E. Kadoglou

**Affiliations:** Medical School, University of Cyprus, Nicosia 2029, Cyprus; nikoskad@yahoo.com or kadoglou.nikolaos@ucy.ac.cy

Cardiovascular diseases (CVDs) remain the leading cause of morbidity and mortality worldwide, with high social–economic costs. Intensive research is being carried out in this field to prevent the development and the consequences of CVDs. This Special Issue, titled “Drug Candidates for the Prevention and Treatment of Cardiovascular Diseases”, aims to provide evidence about the efficacy of novel pharmaceutical and non-pharmaceutical agents in primary and secondary prevention of CVDs (contributions 1 and 2). Moreover, this Special Issue includes studies providing an overview of the cardioprotective properties of established pharmaceutical medications usually prescribed in other diseases, some of them beyond their initial indications, which can be useful to reduce the global cardiovascular burden [1,2].

The eighteen articles published in this Special Issue highlight different aspects of the prevention and treatment of CVDs. The link between inflammatory conditions and CVDs [3] may have several mechanistic explanations (contribution 3). Among them, psoriasis is a good example of auto-immune diseases predisposing individuals to CVDs, where novel biological agents may become the proper candidates for prevention [4]. Ikonomidis I et al. (2023) examined the phosphodiesterase 4 inhibitor apremilast in patients with psoriasis and showed that it had greater efficacy than etanercept or cyclosporine with regard to improving vascular and myocardial function after 4 months (contribution 4). Vasodilatory action and reduced vascular damage after the administration of fetal hemoglobin inducer agent 3-(1,3-dioxoisoindolin-2-yl) benzyl nitrate (Lapdesf-4c) was demonstrated in the aorta rings of Wistar rats, implicating a potential role in patients with endothelial dysfunction, such as those with sickle cell anemia (contribution 5).

The pleiotropic mechanisms of statins have long been investigated and play a significant role in CVD prevention [5]. Two important articles in this Special Issue investigated the impact of lipid-lowering agents on atherosclerosis. In particular, C-6′a-hydroxymethyl monacolin J, with a greater ability to inhibit HMG-CoA reductase, could be a new drug candidate for dyslipidemia treatment (contribution 6). In the case of failure to achieve lipid targets or when statin therapy is intolerable, Proprotein Convertase Subtilisin/Kexin Type 9 (PCSK9) inhibitors may be effective lipid-lowering agents in patients with established CVD or at high risk for CVD [6]. PCSK-9 inhibitor therapy can modify proinflammatory cytokine and matrix metalloproteinase release in patients with mixed hyperlipidemia and vulnerable atherosclerotic plaque (contribution 7). The latter' implies that it has stabilizing effects in atherosclerotic CVDs.

A significant number of articles in this Special Issue examined the effects of new drug candidates on cardiac function. Schnaubelt S et al. studied landiolol, a highly β1-selective beta-blocker, in critically ill patients hospitalized in intensive care units (contribution 8). That drug has been recognized as a well-tolerated, safe and effective β-blocker, especially recommended in acute conditions [7]. In patients diagnosed with STEMI undergoing coronary angioplasty, the incubation of H9c2 cells with exosomes reperfused with citicoline favorably modified the expression of specific miRNAs and proteins related to cardioprotection (contribution 9). In this context, a selective, high-affinity, eight-amino-acid peptide inhibiting cardiac troponin I interaction and phosphorylation by protein kinase C delta (δPKC) may prevent myocardial tissue ischemia/reperfusion injury in a Langendorff model of myocardial infarction ex vivo (contribution 9). Irisin, a novel myokine associated with exercise benefits, is encoded by the FNDC5 (fibronectin type-III domain-containing 5) gene. Continuous irisin pump infusion implanted in Sprague Dawley rats for 42 days ameliorated numerous genes involved in cardiac physiology—FNCD5, Raf1, CPT1, IGF-1, CALCIN, PGC1, Nox4 and Mfn1—promoting cardiac function (contribution 10). Finally, in the field of herbal medicine, limited data, mostly from experimental studies, support the atheroprotective effects of quercetin and silibinin/silymarin (contribution 2).

In light of these diverse contributions, multidisciplinary researchers may gain inspiration from this Special Issue to further study all the potential drug candidates in pre-clinical- and clinical-level studies and draw firm conclusions about their cardiovascular protective effects.
